# Variations in tuberculosis prevalence, Russian Federation: a multivariate approach

**DOI:** 10.2471/BLT.19.229997

**Published:** 2019-09-03

**Authors:** Ivan Meshkov, Tatyana Petrenko, Olivia Keiser, Janne Estill, Olga Revyakina, Irina Felker, Mario C Raviglione, Vladimir Krasnov, Yakov Schwartz

**Affiliations:** a FSBI Novosibirsk Tuberculosis Research Institute, Ministry of Health of the Russian Federation, Novosibirsk, Okhotskaya street 81A, 630040, Russian Federation.; b Institute of Global Health, University of Geneva, Geneva, Switzerland.; c Global Health Centre, University of Milan, Milan, Italy.

## Abstract

**Objective:**

To analyse the epidemiological trends of tuberculosis in the Siberian and Far Eastern federal districts, the areas with the highest disease burden in the Russian Federation.

**Methods:**

We applied principal coordinate analysis to study a total of 68 relevant variables on tuberculosis epidemiology, prevention and control. Data on these variables were collected over 2003–2016 in all 21 regions of the Siberian federal district and Far Eastern federal district (total population: 25.5 million) through the federal and departmental reporting system. We identified the regions with a favourable or unfavourable tuberculosis epidemiological profile and ranked them as low or high priority for specific interventions.

**Findings:**

The median number of tuberculosis notifications in the regions was 123.3 per 100 000 population (range: 54.5–265.7) in 2003, decreasing to 82.3 per 100 000 (range: 52.9–178.3) in 2016. We found large variations in the tuberculosis epidemiological profile across different regions. The principal coordinate analysis revealed that three aggregated indicators accounted for 55% of the variation. The first coordinate corresponded to tuberculosis prevalence and case notifications in the regions; the second to the severity of the disease among patients; and the third to the percentage of multidrug-resistant tuberculosis among tuberculosis patients. The regions where intervention was most urgently needed were Chukotka Autonomous Okrug, Jewish Autonomous Oblast and Tyva Republic.

**Conclusion:**

The variability in tuberculosis epidemiology across regions was likely due to differences in the quality of antituberculosis services. Precision in defining necessary interventions, as determined through the principal coordinate analysis approach, can guide focused tuberculosis control efforts.

## Résumé

**Objectif:**

Analyser les tendances épidémiologiques de la tuberculose dans le district fédéral sibérien et le district fédéral extrême-oriental, qui présentent la charge de morbidité la plus élevée au sein de la Fédération de Russie.

**Méthodes:**

Nous avons appliqué une analyse en coordonnées principales pour étudier un total de 68 variables pertinentes sur l'épidémiologie, la prévention et le contrôle de la tuberculose. Des données sur ces variables ont été collectées entre 2003 et 2016 dans les 21 régions du district fédéral sibérien et du district fédéral extrême-oriental (population totale: 25,5 millions) à l'aide du système de notification fédéral et ministériel. Nous avons déterminé les régions présentant un profil épidémiologique de la tuberculose favorable ou défavorable, puis nous les avons assimilées à un degré de priorité faible ou élevé concernant des interventions spécifiques.

**Résultats:**

Le nombre médian de notifications de cas de tuberculose dans les régions considérées s'élevait à 123,3 pour 100 000 habitants (étendue: 54,5-265,7) en 2003, puis diminuait pour atteindre 82,3 pour 100 000 habitants (étendue: 52,9-178,3) en 2016. Nous avons constaté d'importantes variations du profil épidémiologique de la tuberculose entre les différentes régions. L'analyse en coordonnées principales a révélé que trois indicateurs agrégés contribuaient pour 55% aux variations. La première coordonnée correspondait à la prévalence et aux notifications de cas de tuberculose dans les régions considérées; la deuxième correspondait à la gravité de la maladie chez les patients atteints; et la troisième correspondait au pourcentage de tuberculose multirésistante chez les patients atteints. Les régions où les interventions étaient les plus urgentes étaient le district autonome de Tchoukotka, l'oblast autonome juif et la République de Touva.

**Conclusion:**

La variabilité de l'épidémiologie de la tuberculose entre les régions était probablement due à des différences dans la qualité des services antituberculeux. Il peut être utile de définir précisément les interventions nécessaires, comme déterminé à l'aide de la méthode d'analyse en coordonnées principales, pour orienter les efforts de lutte contre la tuberculose.

## Resumen

**Objetivo:**

Analizar las tendencias epidemiológicas de la tuberculosis en los distritos federales de Siberia y Extremo Oriente, las zonas con mayor carga de morbilidad de la Federación de Rusia.

**Métodos:**

Se aplicó el análisis de coordenadas principales para estudiar un total de 68 variables relevantes en epidemiología, prevención y control de la tuberculosis. Los datos sobre estas variables se recopilaron entre 2003 y 2016 en las 21 regiones del distrito federal de Siberia y del distrito federal del Extremo Oriente (población total: 25,5 millones) a través del sistema de presentación de informes federal y departamental. Se identificaron las regiones con un perfil epidemiológico favorable o desfavorable de la tuberculosis y se clasificaron como de baja o alta prioridad para intervenciones específicas.

**Resultados:**

El número promedio de notificaciones de tuberculosis en las regiones fue de 123,3 por cada 100 000 habitantes (rango: 54,5-265,7) en 2003, disminuyendo a 82,3 por cada 100 000 habitantes (rango: 52,9-178,3) en 2016. Se encontraron notables variaciones en el perfil epidemiológico de la tuberculosis entre las diferentes regiones. El análisis de las coordenadas principales reveló que tres indicadores agregados representaban el 55 % de la variación. La primera coordenada correspondía a la prevalencia de la tuberculosis y las notificaciones de casos en las regiones; la segunda a la gravedad de la enfermedad entre los pacientes; y la tercera al porcentaje de tuberculosis multirresistente entre los pacientes con tuberculosis. Las regiones en las que la intervención era más urgente eran el Distrito Autónomo de Chukotka, la Provincia Autónoma Hebrea y la República de Tyva.

**Conclusión:**

La variabilidad en la epidemiología de la tuberculosis entre regiones se debió probablemente a las diferencias den la calidad de los servicios de tratamiento antituberculoso. La precisión en la definición de las intervenciones necesarias, determinada a través del enfoque de análisis de coordenadas principales, puede guiar los esfuerzos focalizados de control de la tuberculosis.

## ملخص

**الغرض:**

تحليل الاتجاهات الوبائية للسل في المناطق الفيدرالية في سيبيريا والشرق الأقصى، وهي المناطق التي تمثل أعلى عبء للمرض في الاتحاد الروسي.

**الطريقة:**

قمنا بتطبيق تحليل الإحداثيات الرئيسي لدراسة إجمالي 68 من المتغيرات ذات الصلة، على وباء السل والوقاية منه والسيطرة عليه.تم جمع البيانات حول هذه المتغيرات خلال الفترة من 2003 إلى 2016 في جميع الأقاليم الـ 21 في منطقة سيبيريا الفيدرالية ومناطق الشرق الأقصى الفيدرالية (إجمالي السكان:25.5 مليون نسمة) من خلال نظام الإبلاغ الفيدرالي والإداري.حددنا المناطق ذات الظروف الوبائية المواتية أو غير المواتية للسل، وقمنا بتصنيفها على أنها ذات أولوية عالية أو منخفضة بالنسبة لتدخلات معينة.

**النتائج:**

بلغ متوسط عدد إخطارات الإصابة بالسل في المناطق، 123.3 لكل 100000 نسمة (نطاق القياس:54.5 إلى 265.7) في 2003، وانخفض إلى 82.3 لكل 100000 نسمة (نطاق القياس:52.9 إلى 178.3) في عام 2016. وجدنا اختلافات كبيرة في ظروف وباء السل في المناطق المختلفة.كشف تحليل الإحداثيات الرئيسي أن هناك ثلاثة مؤشرات مجتمعة يعود إليها السبب في 55٪ من الاختلاف.يشير الإحداثي الأول إلى انتشار السل وإشعارات الحالات في المناطق؛ ويشير الإحداثي الثاني لشدة المرض بين المرضى؛ بينما يشير الإحداثي الثالث إلى النسبة المئوية لمرض السل المقاوم للأدوية المتعددة بين مرضى السل.كانت المناطق ذات الاحتياج العاجل جداً للتدخل هي Chukotka Autonomous Okrug، وJewish Autonomous Oblast، وTyva Republic.

**الاستنتاج:**

من المحتمل أن التباين في وباء السل عبر المناطق، كان بسبب المستويات المختلفة لجودة خدمات مكافحة السل. يمكن للدقة في تحديد التدخلات الضرورية، كما هو محدد في نهج تحليل الإحداثيات الرئيسي، أن تؤدي إلى توجيه الجهود المركزة لمكافحة مرض السل.

## 摘要

**目的:**

旨在分析结核病在俄罗斯联邦疾病负担最高的地区（西伯利亚和远东联邦区）内的流行病学趋势。

**方法:**

我们采用主坐标分析法，对结核病流行病学、预防和控制共 68 个相关变量进行研究。2003 年至 2016 年期间通过联邦和部门报告系统在西伯利亚联邦区与远东联邦区的所有 21 个地区收集了变量数据（总人口：2550 万）。我们确定了具有有利或不利结核病流行病学特征的区域并针对特定干预措施将其列为低优先级或高优先级。

**结果:**

2003 年，各区域的结核病通报中位数为 123.3/100000 人（范围：54.5-265.7），2016 年下降到 82.3/100000（范围：52.9-178.3）。我们发现不同地区的结核病流行病学特征存在很大差异。主坐标分析显示，三个综合指标占变量的 55％。第一个坐标对应于各地区的结核病患病率和病例通报；第二个对应于患者的疾病严重程度；第三个对应于结核病患者中耐多药结核病的百分比。最迫切需要干预的地区是楚科奇自治区、犹太自治州和图瓦共和国。

**结论:**

各地区结核病流行病学的差异性可能是由于抗结核病服务的质量不同。通过主坐标分析法确定的必要干预措施精确性可以指导结核病控制工作的集中开展。

## Резюме

**Цель:**

Анализ тенденций развития эпидемиологической ситуации по туберкулезу в Сибирском и Дальневосточном федеральных округах, то есть в областях с максимальным бременем заболевания на территории Российской Федерации.

**Методы:**

Авторы применили анализ главных координат для изучения в общей сложности 68 релевантных факторов эпидемиологической ситуации по туберкулезу, его профилактике и контролю. За период с 2003 по 2016 год были собраны данные по этим факторам в общей сложности в 21 субъекте Сибирского федерального округа и Дальневосточного федерального округа (общая численность населения: 25,5 млн человек) на основе федеральной и ведомственной систем отчетности. Авторы определили регионы с благоприятным и неблагоприятным эпидемиологическим профилем по туберкулезу и классифицировали их по группам с низким и высоким уровнем необходимости специализированного вмешательства.

**Результаты:**

Среднее число уведомлений о заболевании туберкулезом в регионах составило 123,3 на 100 000 населения (диапазон 54,5–265,7) в 2003 году; этот показатель сократился до 82,3 на 100 000 населения (диапазон: 52,9–178,3) в 2016 году. Авторы выявили значительную разницу в факторах эпидемиологического профиля по туберкулезу в разных регионах. Анализ главных координат выявил три сводных показателя, ответственные за 55% случаев различия факторов. Первая координата соответствовала распространенности туберкулеза и уведомлениям о случаях заболевания в регионах, вторая соответствовала тяжести заболевания пациентов, а третья — процентной доле мультирезистентной формы туберкулеза среди пациентов. К числу регионов, требующих наиболее срочного вмешательства, относятся Чукотский автономный округ, Еврейская автономная область и Республика Тыва.

**Вывод:**

Разница в показателях эпидемиологической ситуации по туберкулезу в разных регионах, вероятнее всего, обусловлена разным уровнем качества противотуберкулезных мероприятий. Точное определение необходимых вмешательств, выявленных методом анализа главных координат, может помочь в концентрации усилий по борьбе с туберкулезом.

## Introduction

During the 1990s the tuberculosis epidemic in the Russian Federation worsened due to an economic recession in the country at that time. National tuberculosis case notifications reached a peak of 90.7 per 100 000 in the population of 146.6 million in the year 2000. The robust measures to control tuberculosis taken by the government between 2000 and 2010 led to a reduction in case notifications to 48.3 per 100 000 in the population of 144.5 million by 2017. Despite these measures, the tuberculosis situation in the Asian part of the Russian Federation remained less favourable compared with the European part. For example, in the year 2017, tuberculosis case notifications in the Siberian federal district (population: 19.3 million) and Far Eastern federal district (population: 6.1 million) were as high as 83.3 and 86.1 per 100 000 respectively.^1^^–^^4^ Identification of the key factors that determine the unfavourable epidemic situation in these districts is therefore a top priority for research. The evidence collected can then be used to plan focused interventions.

Tuberculosis surveillance and monitoring were established in their present form in these two federal districts in 2003 with data pm dozens of variables are collected annually by the government. We aimed to identify the variables that are closely correlated and to understand how they interact and contribute to the tuberculosis epidemic in different regions of Siberian and Far Eastern federal districts. We used multivariate principal coordinate analysis,^5^ a method that allowed us to condense the complex, multidimensional data set into a small number of aggregated indicators for each region. Description of the regions’ tuberculosis status in terms of these indicators would enable us to characterize and compare the epidemiology of tuberculosis among regions. The results are intended to inform the development of a unified prevention, care and control policy that addresses each region’s specific needs.

## Methods

### Data source

The study was a retrospective epidemiological analysis covering all tuberculosis cases notified in the Asian part of the Russian Federation from 2003 to 2016. 

We analysed data obtained from federal and department reports for each of the 21 regions in the Siberian and Far Eastern federal districts which constitute the Asian part of the Russian Federation. The health authority reports cover all registered patients with records of all forms of tuberculosis. A total of 68 variables of tuberculosis incidence, treatment and outcomes are collected. The method of data gathering is identical in all regions of both federal districts. Access to the epidemiological data is restricted to clinical specialists and medical statisticians. The raw data are obtained by regional tuberculosis centres from tuberculosis hospitals and outpatient departments at regional, municipal and township levels using official ministerial guidelines, and collated for analysis in Novosibirsk Tuberculosis Research Institute. The data are regularly checked for consistency and reliability by experienced research personnel at the Institute who frequently conduct supervisory visits in the observed regions.

A detailed description of all variables collected by the federal authorities and their calculation rules and periods of data collection are available from the data repository.^6^ These variables are recommended for epidemiological reporting as key factors in control of epidemic tuberculosis.^7^^,^^8^ Variables cover a wide range of characteristics, including: (i) case notifications and the number of notified tuberculosis cases stratified by age and by urban or rural population; (ii) data on tuberculosis patients who were difficult to treat, including those with multidrug-resistant (MDR) tuberculosis or extensive fibrotic changes and cavities (verified by a medical board of experts reviewing difficult cases using clinical and tomography data);^9^^–^^11^ (iii) coverage of tuberculosis diagnostics, including any type of examination; and (iv) outcomes of tuberculosis treatment.

### Data analysis

We used principal coordinate analysis for the study. In this method, the differences between regions are represented by a small number of coordinates. The method has several advantages compared with more commonly used methods such as principal component analysis. First, principal coordinate analysis has low sensitivity to the presence of outliers in the data. Second, the method can reflect the relationships between the variables analysed regardless of whether they are linear or not. Third, it has good applicability when the number of variables (in our case the epidemiological data) is much greater than the number of observations (in our case the different regions). 

We first compiled a table containing the values of all the variables, by year and region. We prepared the table for principal coordinate analysis by transforming the initial data to standardize the scatter of the analysed variables.^12^^,^^13^ Without these transformations, the effect of variables with relatively small variances would contribute insufficiently to the analysis. For this purpose, we calculated the median values and interquartile ranges for all variables of the initial data and then converted the data using the formula:
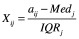
(1)Where *a_ij_*is the initial value of variable *j* for the region *i, Medj* is a median value and the *IQR_j_*interquartile range of the variable *j* over all regions, and *x_ij_* is the final transformed value.

All collected variables should be used to achieve a comprehensive description of the difference in the epidemic situation between the regions. It is possible to choose a suitable metric, then to calculate the differences (called distances) between regions and finally to construct the matrix of distances. For the present study we chose Manhattan distances and calculated the distances between the regions using the formula:
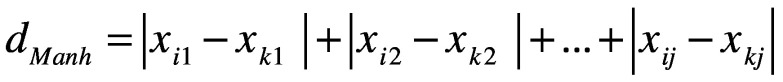
(2)where *i* and *k* are the regions between which the distance is calculated, and 1, 2, … *j* are the variables. We chose this metric because the values of variables for pairwise correlation of two regions were mostly identical or slightly different, but there were several variables with large differences of values. In this case, the Manhattan distance will smooth these differences. 

To analyse the matrix of the Manhattan distances, information from the distance matrix is used to form a limited number of new variables (coordinates) that are the measures of (dis) similarity between regions and that explain most of the interregional variability. The regions are visualized as points on the coordinate axes of the scatterplot. Similarity between regions is represented as the simple geometric distance between points. The shorter the geometric distance, the higher the similarity.

We assessed the explanatory value of the principal coordinates using the eigenvalues of each coordinate. We divided the eigenvalues of each coordinate by the sum of the eigenvalues of the entire coordinate set. The data transformation is considered informative if two or three coordinates with relatively large eigenvalues (encompassing more than 50% of the total variance) are obtained, while for other axes the eigenvalues are small. The principal coordinate values we obtained are dimensionless quantities. In our case, it was convenient to present the administrative regions as points on a three-dimensional scatterplot. 

The epidemiological meaning of a coordinate can be understood from the value of its correlation with 68 initial epidemiological variables, according to the epidemiological meanings of these variables. The Spearman correlations between variables and coordinates were calculated for each year of observation. Only strong relationships with correlation coefficients ≥ 0.6 in absolute value were considered.

We used R programming language, version 3.4.0 (R Foundation for Statistical Computing, Vienna, Austria) for statistical analysis.^14^

## Results

### Tuberculosis time trends

**Fig. 1:**
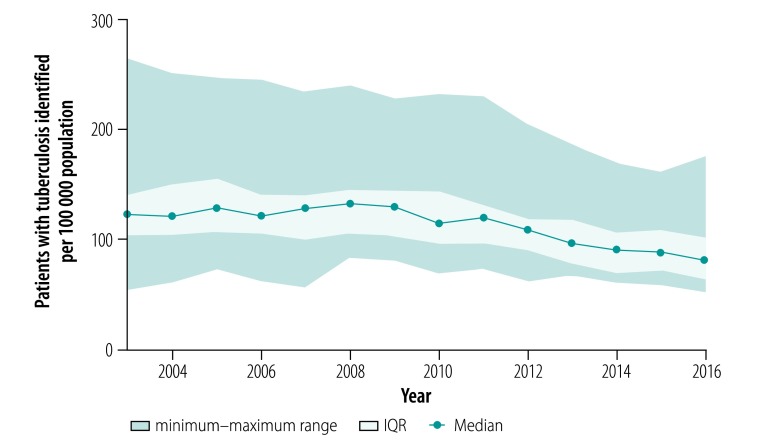
Tuberculosis case notifications in the Asian part of the Russian Federation, 2003–2016

The median number of tuberculosis case notifications was 123.3 per 100 000 population in 2003, ranging from 54.5 to 265.7 per 100 000 across the 21 regions (Fig. 1). Notifications remained somewhat stable up to the year 2010, after which they decreased in most regions, to a median of 82.3 per 100 000 in 2016 (range: 52.9–178.3 per 100 000).

IQR: interquartile range.

Notes: We analysed data obtained from federal and department reports. We calculated median values of tuberculosis case notifications as the ratio of all tuberculosis cases notified for the first time (including postmortem diagnoses) during the year to the mid-year total population of the region.

**Fig. 2:**
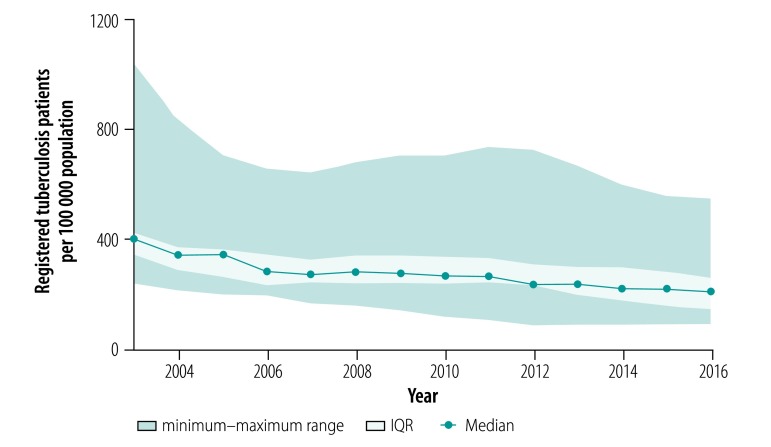
Tuberculosis prevalence in the Asian part of the Russian Federation, 2003–2016

**Fig. 3:**
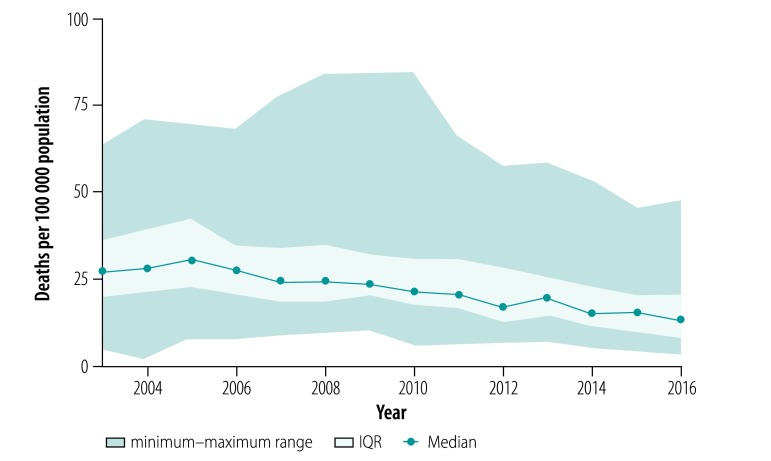
Tuberculosis mortality in the Asian part of the Russian Federation, 2003–2016

**Fig. 4:**
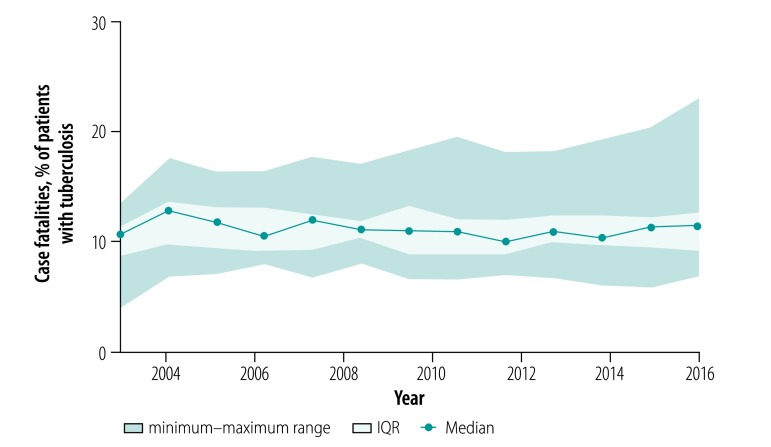
Case fatalities in the Asian part of the Russian Federation, 2003–2016

**Fig. 5:**
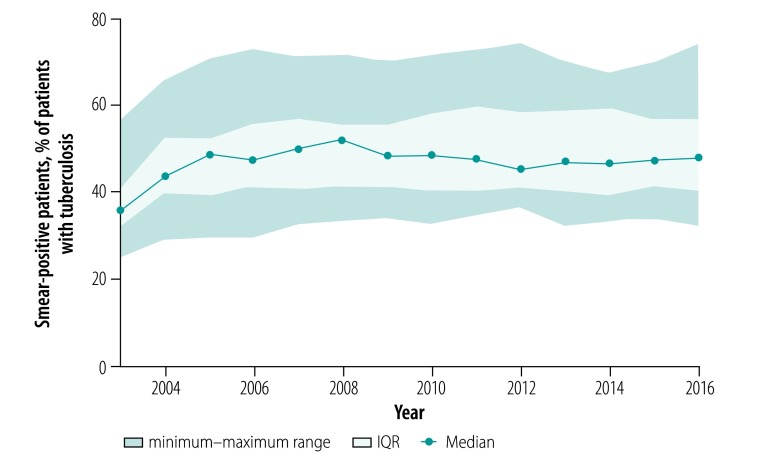
Proportion of smear-positive tuberculosis in all followed patients with respiratory tuberculosis in the Asian part of the Russian Federation, 2003–2016

In the analysis by region, we found that tuberculosis prevalence (Fig. 2) and mortality (Fig. 3) between the years 2003 and 2016 decreased in all regions except Chukotka Autonomous Okrug. Case fatalities among the mid-year average number of tuberculosis patients were stable in all regions during the whole observation period (median: 11.7% in 2016; Fig. 4). The exceptions were in Novosibirsk Oblast, Kemerovo Oblast and Irkutsk Oblast, where case fatalities increased from 11.8% to 16.3%, 18.5% to 23.2% and 15.0% to 17.8%, respectively between 2010 and 2016. The percentage of smear-positive patients among those with respiratory tuberculosis increased in most regions between the years 2003 and 2005 and stabilized thereafter (median: 47.7% in 2016; Fig. 5).

IQR: interquartile range.

Note: We calculated median values of tuberculosis prevalence as the median ratio of followed tuberculosis patients during the year to the mid-year total population of the region.

IQR: interquartile range.

Note: We calculated median values of tuberculosis mortality as the ratio of all registered deaths of tuberculosis patients caused by tuberculosis during the year to the mid-year total population of the region.

IQR: interquartile range.

Note: We calculated median values of case fatality as the ratio of all tuberculosis-related deaths among tuberculosis patients during the year to the mid-year average number of tuberculosis patients in the region.

IQR: interquartile range.

Note: We calculated median values of the percentage of smear-positive tuberculosis cases among followed patients with respiratory tuberculosis during the year of observation.

### Principal coordinates

In the principal coordinate analysis, we found that the first three coordinates explained 55.1% of the variability of the initial epidemiological data across regions: the first coordinate explained 28.6% of the variability, the second explained 14.1% and the third explained 12.4%.

#### Epidemiological favourability

The first coordinate correlated inversely with 13 variables, including: tuberculosis case notification by chest X-ray screening examination per 1000 people; tuberculosis case notification in the total population, in rural and in urban populations (as percentages of the total population) and among adolescents aged 15–17 years; tuberculosis prevalence per 100 000 people among adolescents and adults; and tuberculosis relapse and mortality. An inverse relationship was also found between this coordinate and the prevalence of tuberculosis with extensive fibrotic changes and cavities; and the proportion of patients with that type of tuberculosis among all tuberculosis patients. There was a positive correlation with two variables: the percentage of respiratory tuberculosis patients with bacteriological conversion; and the proportion of closure of cavities among tuberculosis patients. The Spearman correlation coefficients and *P*-values of all variables correlated with the first coordinate are available from the data repository.^15^

The first coordinate could be interpreted as an aggregated indicator of interregional variations of epidemiological tuberculosis transmission and severity, and we called it the epidemiological favourability coordinate. The high values of this coordinate reflect both a low level of tuberculosis transmission and a high efficiency of antituberculosis measures. The higher the proportion of cured patients, the lower the relapse rate and the prevalence of tuberculosis.

#### Severe tuberculosis

The second coordinate correlated negatively with six variables, including: tuberculosis case notifications in the total population and in urban areas; the total number of people registered for treatment; the number of followed patients co-infected with HIV and tuberculosis; tuberculosis-related case fatality and mortality; and the proportion of tuberculosis patients who died outside hospitals in 2006, 2011 and 2014. There was a positive correlation of this coordinate with one variable: the density of tuberculosis specialists in the region. The Spearman correlation coefficients of all variables correlated with the second coordinate are available from the data repository.^16^

The correlations indicate that the regions characterized by high values of the second coordinate were those with a relatively low prevalence of severe tuberculosis. Moreover, these relationships probably indicate that a high concentration of tuberculosis specialists in a region increased the cure rate and lowered tuberculosis related mortality. The second coordinate therefore reflects the interregional variations in the efficacy of interventions in preventing severe tuberculosis and in reducing mortality and could be called the control of severe tuberculosis coordinate. This coordinate correlated inversely with tuberculosis case notifications in urban populations, but no relationship was found in rural populations. This finding can probably be explained by the higher availability of antituberculosis services and the higher number of tuberculosis specialists in the cities.

#### MDR tuberculosis

The third coordinate correlated negatively with seven variables, including: the proportions of MDR tuberculosis cases among tuberculosis patients; MDR tuberculosis case notifications; and the percentage of cavity closures in newly diagnosed patients. This coordinate was therefore associated with the control of MDR tuberculosis. The detailed list of variables correlated with the third coordinate is available from the data repository.^17^

The data show that the more patients newly registered for treatment, the higher the proportion of diagnosed cases with MDR tuberculosis. Between the years 2005 and 2010, the tuberculosis epidemic was particularly severe in certain regions, which prompted government-supported interventions. The measures included additional financing, strengthening of tuberculosis services, more training courses for tuberculosis specialists, an increased number of radiological examinations and an increase in the number of tuberculosis surgeries. These actions may have led to an increase in the number of diagnosed bacteriological conversions with a subsequent decrease in transmission and, ultimately, tuberculosis notifications and mortality after 2010. However, we cannot exclude that during these interventions some patients were still treated inadequately, leading to low cure rates and an increased number of patients with MDR tuberculosis.^18^

### Geographical variations

**Fig. 6:**
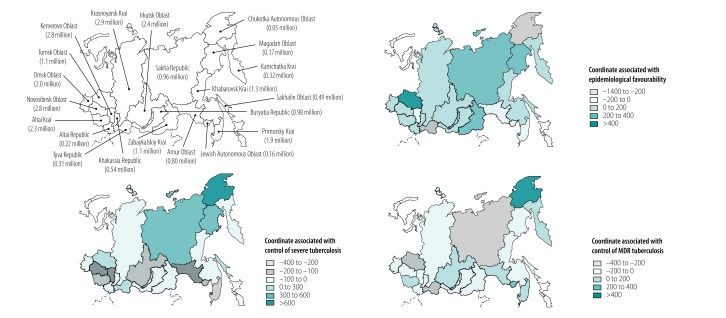
Distribution of the three principal coordinates of tuberculosis epidemiology in 21 regions of the Asian part of the Russian Federation, 2003–2016

**Fig. 7:**
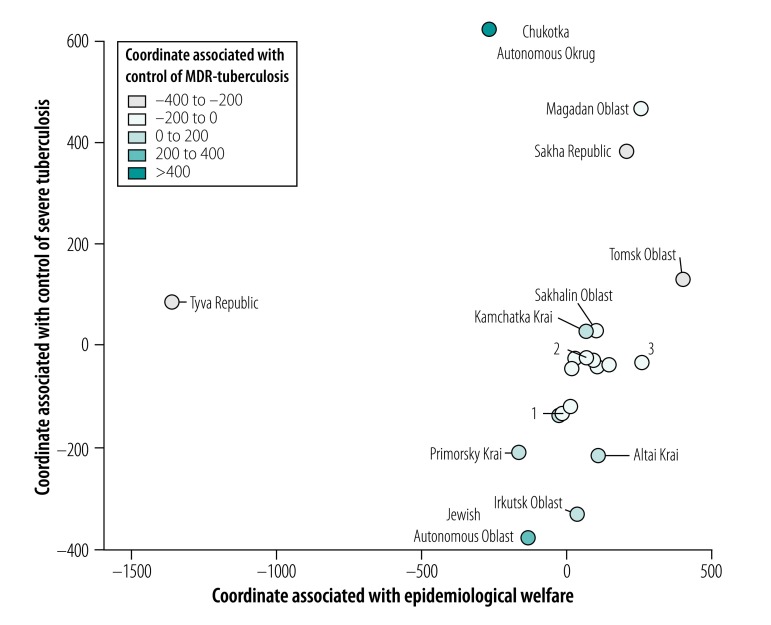
Location of administrative regions in the Asian part of the Russian Federation in three principal coordinates in accordance with the values of tuberculosis epidemiological variables

The values of the three coordinates for all regions are shown on a map of the Russian Federation (Fig. 6) and three-dimensional scatter plot (Fig. 7; available at http://www.who.int/bulletin/volumes/97/11/19-229997). Of the 21 regions, Tomsk Oblast had the highest value for the first coordinate, associated with epidemiological welfare. The lowest values of the epidemiological welfare coordinate were in Tyva Republic and Chukotka Autonomous Okrug. The value of the second coordinate, associated with control of severe tuberculosis, was highest in Chukotka Autonomous Okrug and lowest in Jewish Autonomous Oblast, Irkutsk Oblast, Altai Krai and Primorskiy Krai. The value of the third coordinate, associated with control of MDR tuberculosis, was also highest in Chukotka Autonomous Okrug region, but lowest in Sakha Republic, Tyva Republic and Tomsk Oblast.

MDR: multidrug resistant.

Notes: Administrative regions constitute Siberian and Far East Federal districts of the Russian Federation. Regions are shaded according to the values of the three principal coordinates; darker shades of colour indicate a higher value of the coordinate. The principal coordinate values are dimensionless quantities. Population data is from 2016.

Source: These maps are derivatives of the map presented in the ReliefWeb library.^19^

MDR: multidrug resistant.

Notes: The administrative regions are represented as points plotted accordingly to their values on the principal coordinates. The values of the epidemiological welfare and control of severe tuberculosis coordinates correspond to the x- and y-axes respectively and the values of the multidrug-resistant tuberculosis control coordinate are shown by the white–black gradient of the points. For positive correlations, the higher the value of the principal coordinate, the higher the value of associated epidemiological variables. For negative correlations, the higher the value of the principal coordinate, the lower the value of associated epidemiological variables. Groups of administrative regions are coded as follows: 1. Amur Oblast, Kemerovo Oblast and Novosibirsk Oblast; 2. Altai Republic and Buryatia Republic, Khabarovsk Krai, Khakassia Republic, Krasnoyarsk Krai and Omsk Oblast; 3. Zabaykalsky Krai.

The prevalence and incidence of tuberculosis in Chukotka Autonomous Okrug and Tyva Republic over the study period are available from the data repository.^20^^,^^21^ Chukotka Autonomous Okrug was the only region with a steady increase in tuberculosis case notifications and prevalence during 2008–2016. Tyva Republic had the highest tuberculosis prevalence and case notifications over the whole period of observation.

Tuberculosis case notifications in the urban populations of the Jewish Autonomous Oblast, Irkutsk Oblast, Altai Krai and Primorskiy Krai regions were higher over the years 2010–2016 than in most of other regions (available from the data repository).^22^ These four regions are characterized by their markedly low values of the second coordinate and thereby unfavourable profile of severe tuberculosis. Irkutsk Oblast had the highest prevalence of HIV and tuberculosis co-infection between 2010 and 2015 (available from the data repository).^23^ The Jewish Autonomous Oblast had high tuberculosis mortality from 2004 (available from the data repository).^24^

## Discussion

We found large variations in the epidemiology of tuberculosis across different regions of the Asian part of the Russian Federation. Using principal coordinate analysis, we could identify regions with a more favourable tuberculosis epidemiological and control situation. The more favourable epidemiological profile of tuberculosis in Tomsk Oblast may be attributed to the drug-resistant tuberculosis programme, which was implemented only in this region. The programme was financed by the Global Fund to Fight AIDS, Tuberculosis and Malaria according to WHO recommendations, implemented between 2000 and 2013. The programme has been recognized as one of the most successful projects to manage MDR tuberculosis in the world.^25^

We could also rank regions as high or low priority for specific interventions. The heterogeneity observed can be explained mainly by differing levels of antituberculosis services in different regions. Each region in this part of Russian Federation has its own specific characteristics and needs specific interventions to improve the situation. The regions where intervention is most urgently needed were Chukotka Autonomous Okrug, Jewish Autonomous Oblast and Tyva Republic. Although tuberculosis prevalence, notification and mortality rates have decreased in Tyva Republic over the past 14 years, the general situation of tuberculosis control in this region was unfavourable compared with other regions. In Chukotka Autonomous Okrug during 2011–2016, tuberculosis case notifications in the rural population were 3.0–5.7 times higher than in the urban area, which consists only of Anadyr town. In rural areas this variable had a range of 226.7–373.1 per 100 000 population from 2011 to 2016 versus 63.6–86.2 per 100 000 population in the urban area. In rural areas, where most of the population is of indigenous ethnicity, medical support is difficult to access. Patients from indigenous populations tend to seek medical care late during the disease and often after developing advanced tuberculosis lesions. These patients often then become a source of transmission within the community. Factors promoting a steady increase of tuberculosis notifications include the crowded living conditions in some traditional houses (*yarangas*) and insufficient access to health care.^26^ The high tuberculosis mortality and case fatality in Jewish Autonomous Oblast can be explained by the presence of the Federal Penitentiary Service interregional tuberculosis hospital. This hospital serves tuberculosis patients from prisons located not only in this region, but also from neighbouring regions. All deaths in this hospital are of prisoners, thereby increasing the overall regional tuberculosis mortality by 15–20%.^27^

We found low values on the coordinate associated with control of severe tuberculosis in Irkutsk Oblast and Jewish Autonomous Oblast. These data demonstrate the need to improve tuberculosis detection and treatment, to address the issue of HIV and tuberculosis co-infection in Irkutsk Oblast and to decrease tuberculosis deaths in the prison hospital in Jewish Autonomous Oblast.

The high values of MDR tuberculosis control in Chukotka Autonomous Okrug and Jewish Autonomous Oblast do not imply a low burden of MDR tuberculosis, but rather reflect a lack of capacity for diagnosing these patients. These were the only regions in which tuberculosis facilities were not equipped with the Xpert® *Mycobacterium tuberculosis*/rifampicin (MTB/RIF) rapid automated nucleic acid amplification assay (Cepheid, Sunnyvale, United States of America) until the year 2016. The Xpert MTB/RIF assay was gradually implemented in most regions during the years 2013–2016, which could potentially bias the data on variables related to MDR tuberculosis. However, the proportion of multidrug resistance among all patients with tuberculosis increased during this period in only two regions (Irkutsk Oblast and Altai Krai) and thus the risk of bias is unlikely. The low rates of reported MDR tuberculosis before the implementation of the Xpert MTB/RIF assay in Irkutsk Oblast and Altai Krai are likely explained by the previous poor rates of detecting MDR tuberculosis.

The high levels of MDR tuberculosis in Sakha Republic and Tomsk Oblast can be attributed to the high quality of tuberculosis diagnostic services in these regions. In contrast, in Tyva Republic, high numbers of multidrug-resistant infections may be explained by inadequate treatment and compliance, and inadequately qualified medical personnel.^28^^,^^29^ If the quality of diagnostic services in Tyva were improved, the multidrug-resistance levels would likely be even higher. Indeed, in the years 2011–2016 the proportion of smear-positive patients who were tested for drug sensitivity in the Tyva Republic varied between 44.8% and 67.8%, whereas in Tomsk Oblast and Sakha Republic the proportion was above 85%. Of note, Tyva Republic had the highest tuberculosis prevalence and case notifications among all 21 regions. 

This study has some limitations. We included variables in the analysis that were measured longitudinally, but each value was considered independently; we could therefore not describe the time trends of the variables using principal coordinate analysis. Since we did not have data at the individual level, correlations between variables could also be marginally affected by some unmeasured confounders. As this work focused on a large geographical area with a high burden of tuberculosis, specific population subgroups, such as marginalized communities or inhabitants from hard-to-reach rural areas, may not be completely covered by the analysis. However, the proportion of these people in the total population is small and we believe that the potential bias is negligible.

The overall burden of tuberculosis in the Asian part of the Russian Federation is still serious. The quality of diagnosis and treatment, and the availability of qualified medical personnel operating in properly equipped facilities are the most important factors that can improve individual care and population control of tuberculosis. Precision in defining and implementing the necessary interventions, as defined through the principal coordinate analysis approach, can guide efforts to control tuberculosis in this vast territory.
